# Tinea capitis: dermoscopy and calcium fluorescent microscopy as highly efficient and precise diagnostic tools^☆^

**DOI:** 10.1016/j.abd.2019.06.013

**Published:** 2020-03-19

**Authors:** Hui Xiao, Sushmita Pradhan, Xin Ran, Yuping Ran

**Affiliations:** Department of Dermatovenereology, West China Hospital, Sichuan University, Chengdu, Sichuan, 610041, China

**Keywords:** Dermoscopy, Fluorescent dyes, Microscopy, fluorescent, Tinea capitis

## Abstract

Tinea capitis comprising of tinea favosa and kerion is mostly seen in school-aged children. Some tinea capitis often presented with insignificant findings under the naked eyes are easily overlooked. The authors describe an unusual case of tinea capitis caused by *Trichophyton violaceum*. The patient was an 8-year-old girl, with a history of pruritus on the scalp for more than one year. A diagnosis of tinea capitis was confirmed by clinical examination aided by dermoscopy, calcium fluorescent microscopy and culture. Comma and corkscrew hairs are two specific dermoscopic patterns of tinea capitis. The patient was treated with systemic itraconazole, topical application with 1% naftifine 0.25% ketoconazole cream followed after daily hair wash with 2% ketoconazole shampoo for 8 weeks.

## Introduction

Tinea capitis comprising of tinea favosa and kerion is mostly seen in school-aged children. The incidence of unusual findings of tinea capitis caused by anthropophilic dermatophytes, such as *Trichophyton violaceum*, has grown in recent years. These fungi tend to cause scarce inflammatory reactions, presented with insignificant findings under the naked eyes are easily overlooked. The diagnosis of less symptomatic and atypical cases of tinea capitis is based on the results of direct microscopic examination with conventional 10% KOH smear and mycological cultures of skin scrapings and hair debris. The absence of a rapid and reliable confirmatory test, coupled with a nonspecific presentation, can lead to delayed diagnosis or misdiagnosis. It has been reported that comma and corkscrew hairs are two specific dermoscopic patterns of tinea capitis.^1^, ^2^ Calcium fluorescent microscopy can definitely confirm a diagnosis. Here, we report a case of tinea capitis timely diagnosed with dermoscopy and calcium fluorescent microscopy as highly efficient and precise diagnostic tools.

## Case report

**Figure 1:**
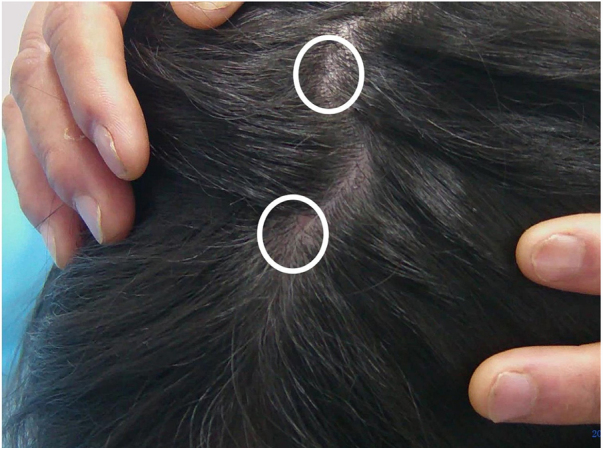
Clinical manifestation of tinea capitis. A small local bean sized hair loss patch and scattered “black spots” on the top of the head (white circles).

**Figure 2:**
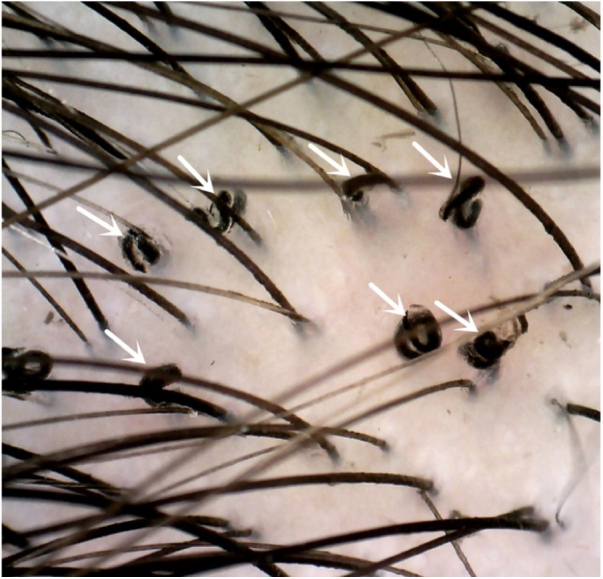
Dermoscopy showed corkscrew hairs (white arrows, original magnification ×40).

**Figure 3:**
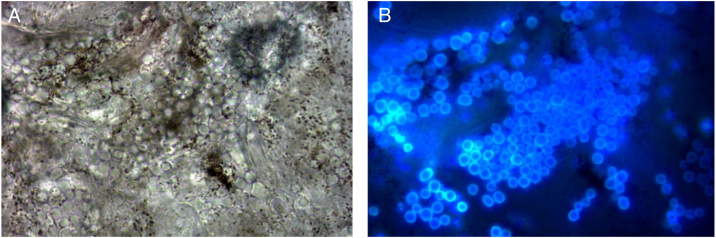
Calcium fluorescent microscopy of scales showed extremely high numbers of spores (original light (A) and fluorescent light (B) in the same field. original magnification ×1000).

**Figure 4:**
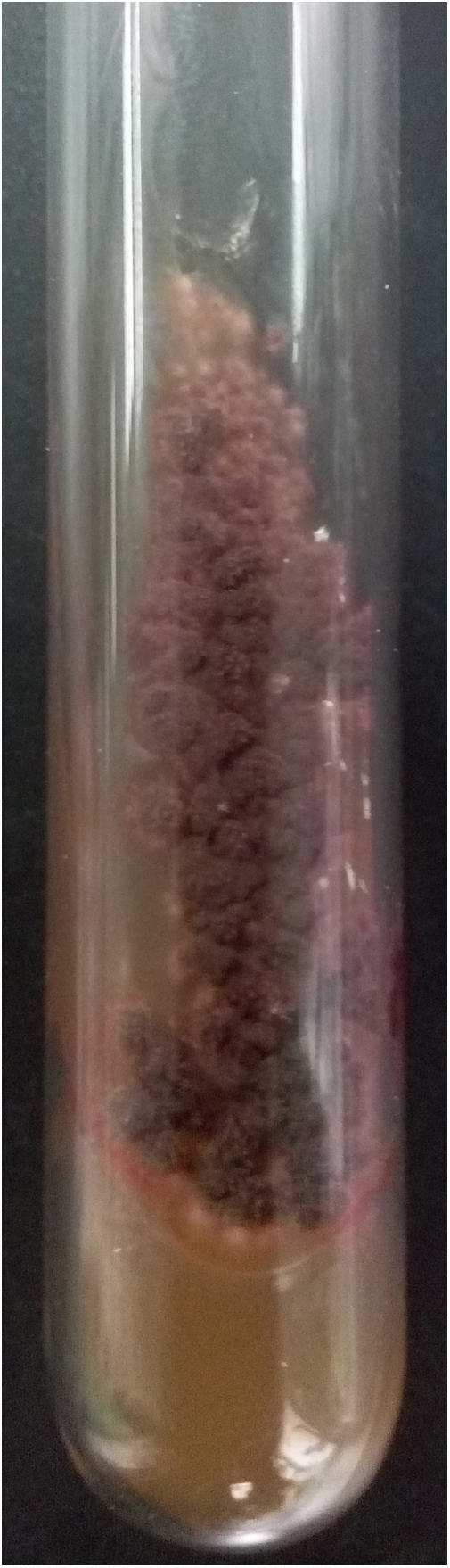
Lush purple colony developed after inoculating the scales in SDA medium at 28 °C for 30 days.

**Figure 5:**
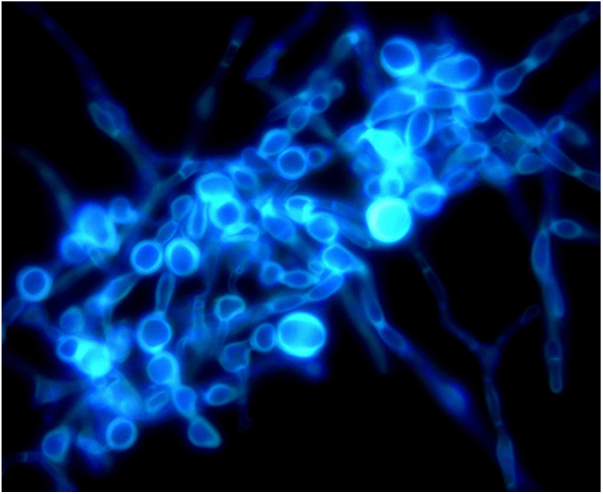
Calcium fluorescent microscopic examination of the smear of colony showed separate branches of mycelium with irregular protrusions, and thick-walled spores of varying sizes after the colony were cultured in PDA medium at 25 °C for 14 days (original magnification ×1000).

**Figure 6:**
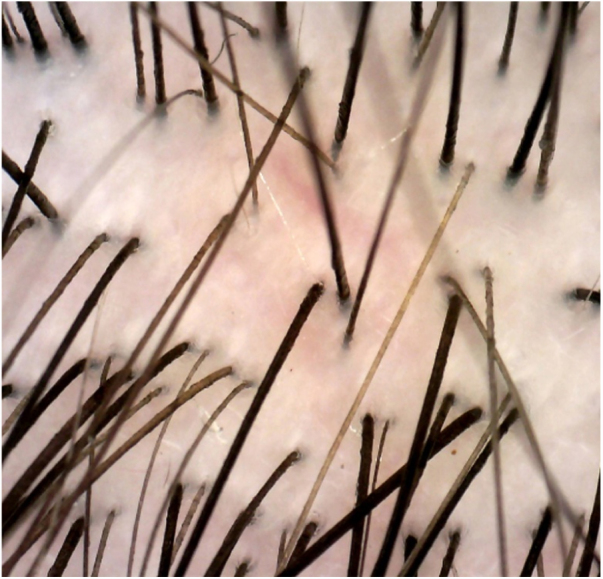
Dermoscopy of post-treatment findings showed the disappearance of corkscrew hairs (original magnification ×40).

An 8-year-old Chinese girl weighing 21 kg presented to the dermatology clinic with a history of pruritus on the scalp for more than one year. Her past medical history was unremarkable. She had a history of taming a cat and a dog at home. Dermatological examination revealed a small bean-sized patch hair loss and scattered “black spots” on the top of the head (Fig. 1). Numerous short, highly convoluted, coiled, and twisted corkscrew hairs were observed under polarized dermoscopy (JD801D; JEDA, China) (Fig. 2). Calcium fluorescent (Fungal Fluorescence Detection Kit, Jiangsu Lifetime Biological Technology Co., Ltd.) microscopy of the scale in corkscrew hair revealed extremely high numbers of spores (Fig. 3). The lush purple colony developed after inoculating the scales in SDA medium at 28 °C for 30 days (Fig. 4). Calcium fluorescent microscopic examination showed separate branches of mycelium with irregular protrusions, and thick-walled spores of varying sizes after the colony were cultured in PDA medium at 25 °C for 14 days (Fig. 5). The girl was diagnosed with an unusual case of tinea capitis caused by *T. violaceum* confirmed by dermoscopy, calcium fluorescent microscopic examination and culture. She received systemic treatment with itraconazole (Xian-Janssen Pharmaceutical Ltd.), 100 mg per day with full fatty milk, combined with topical application of 1% naftifine 0.25% ketoconazole cream (Chongqing Huapont Pharmaceutical Co., Ltd.) followed after daily hair wash with 2% ketoconazole shampoo (Triatop, Xian-Janssen Pharmaceutical Ltd.). The patient showed drastic improvement after 8 weeks of treatment with the disappearance of corkscrew hairs. Therefore, no recurrence was observed after one year of follow-up (Fig. 6).

## Discussion

The previous study speculated the formation of corkscrew hairs as a result of a combination of internal damage due to unsymmetrical hair degradation by *T. violaceum* and external resistance due to scales covering the hair.^3^

Dermoscopy is a diagnostic tool for the identification of fine structures and colors that cannot be observed with the naked eye. It can facilitate the diagnosis of tinea capitis as a rapid, noninvasive, reliable, and an inexpensive method. Calcium fluorescent microscopy is easy to operate and timely that accurately identifies the fungal infections by specific staining of the fungal cell wall. Thus, it can significantly improve its positive rate, on special populations, of children, who can receive early and effective treatment after the rapid diagnosis.^4^

## Financial support

None declared.

## Authors’ contributions

Hui Xiao: Statistic analysis; approval of the final version of the manuscript; conception and planning of the study; elaboration and writing of the manuscript; obtaining, analysis, and interpretation of the data; effective participation in research orientation; intellectual participation in the propaedeutic and/or therapeutic conduct of the studied cases; critical review of the literature; critical review of the manuscript.

Sushmita Pradhan: Statistic analysis; approval of the final version of the manuscript; conception and planning of the study; elaboration and writing of the manuscript; obtaining, analysis, and interpretation of the data; effective participation in research orientation; intellectual participation in the propaedeutic and/or therapeutic conduct of the studied cases; critical review of the literature; critical review of the manuscript.

Xin Ran: Elaboration and writing of the manuscript; obtaining, analysis, and interpretation of the data; effective participation in research orientation; intellectual participation in the propaedeutic and/or therapeutic conduct of the studied cases; critical review of the manuscript.

Yuping Ran: Statistic analysis; approval of the final version of the manuscript; conception and planning of the study; elaboration and writing of the manuscript; obtaining, analysis, and interpretation of the data; effective participation in research orientation; intellectual participation in the propaedeutic and/or therapeutic conduct of the studied cases; critical review of the literature; critical review of the manuscript.

## Conflicts of interest

None declared.
